# Darier's disease: a new paradigm for genetic studies in psychiatric disorders

**DOI:** 10.1590/S1516-31802000000600011

**Published:** 2000-11-01

**Authors:** Quirino Cordeiro, Daniela Meshulam Werebe, Homero Vallada

**Keywords:** Darier's disease, Depression, Psychiatry, Genetics, Doença de Darier, Depressão, Psiquiatria, Genética

## Abstract

**CONTEXT:**

One strategy for identifying susceptibility genes for common disorders is to investigate Mendelian diseases, cosegregating with these common disease phenotypes.

**CASE REPORT:**

A family with seven members is described, in which three members present Darier's disease and depression. This apparent cosegregation, if true, would support the hypothesis that in some pedigrees, a gene for mood disorder may be located on chromosome 12.

## RESUMO

**CONTEXTO:**

Uma estratégia para identificar genes de susceptibilidade para doenças comuns é investigar a co-segregação entre essas enfermidades e algumas doenças mendelianas.

**RELATO DO CASO:**

É descrita uma família com sete membros, dos quais três deles apresentam depressão e doença de Darier. Essa aparente co-segregação, se verdadeira, daria suporte à hipótese que, em algumas famílias, um gene relacionado ao transtorno do humor poderia estar localizado no cromossomo 12.

## INTRODUCTION

Darier's disease (follicular keratosis) is an autosomal dominant genetic disorder, characterized by keratotic plates on the skin. There are descriptions of neuropsychiatric manifestations such as mental deficiency, epilepsy, psychosis and mood disorders in patients with this dermatological disease.^1^ The prevalence of Darier's disease is estimated at 1 per 100,000 inhabitants, with an incidence of 4 new cases per million, over 10 years. The gene which causes the disease was recently identified on chromosome 12 (12q23-q24.1).

## CASE REPORT

A 22 year-old male patient, with a history of Darier's disease since the age of 18, started to present a lack of interest in his activities, low self-esteem, social isolation, terminal insomnia and depressed mood. Within a few weeks the symptoms got worse, and he also developed auditory hallucinations and delusional ideas that were congruent to his mood, i.e. he believed that his neighbors wanted to kill him because he was a despicable human being. Apparently, these psychiatric symptoms were not associated with the worsening of his dermatological symptoms.

He was treated with sertraline at a dose of 150 mg and 2 mg of risperidone per day, with full remission of the psychiatric manifestations. The patient had no previous psychiatric disorder, or any history of abnormal psychosocial behavior. He had had four years of primary school education, and he had been working as an office assistant since the age of 18.

**Figure 1:**
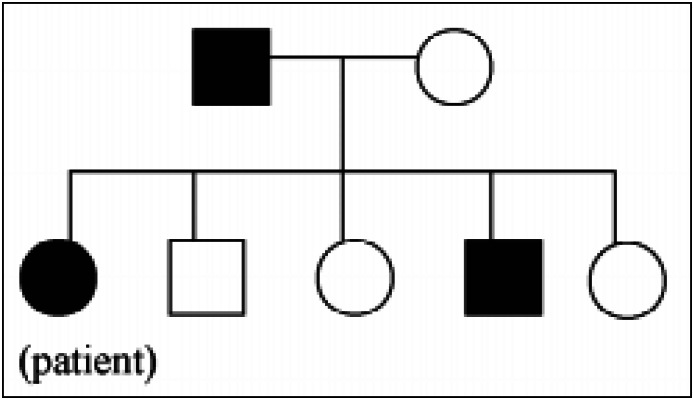
Schematic representation of the family. The black circles and squares are the family members with Darier's disease and depression.

Additionally, his father also presented Darier's disease and showed recurrent major depression but without psychotic symptoms. Another family member, one of the patient's sisters, also had Darier's disease and recurrent major depression without psychotic symptoms. The patient's mother and his other three siblings presented neither a dermatological disease nor any psychiatric disorder *(Figure 1)*.

## DISCUSSION

Since the 19^th^ century, psychiatrists have observed that some psychiatric disorders, like schizophrenia or manic-depressive illness, seem to be more prevalent in some families. This speculative inheritance component has been confirmed by family, twin and adoption studies, with the next step being to identify its gene(s).

One of the strategies used for identifying susceptibility genes for common disorders (also called complex disorders because they are very likely to be etiologically heterogeneous) is to study their cosegregation with a Mendelian disorder. A successful example in psychiatry was the discovery of a Mendelian cause of Alzheimer's disease, located on chromosome 21 (mutations at the APP locus), due to the observation that Down syndrome patients show similar Alzheimer neuropathological features.

The family described above presents co-occurrence of depression and Darier's disease in some of its members. Possible interpretations for this association are:

1. This phenomenon could occur because the gene of Darier's disease also predisposes towards depression (also called the pleiotropic effect). There are previous reports of depression and suicidal behavior in patients with Darier's disease. In addition, skin and brain are originated from the ectoderm, sharing the same embryonic origin. Therefore, it can be speculated that the defect in cellular adhesion expressed in the skin and brain is promoted by an alteration on the same gene. However, the only systematic study of Darier's disease concluded that there was no statistically significant association with psychiatric disorders.^1^
2. Darier's disease, as a chronic dermatological disorder, could promote depressive illness as a psychological stressor. However, Craddock, et al.^2^ reported that those patients with both disorders do not relate their depressive symptoms to the dermatological disease, and apparently the symptoms of each disorder develop at different times. The family presented in this report confirms the observation found in Craddock, et al.^2^ In addition, Burge & Wilkinson^1^ did not find a higher prevalence of depression in patients with Darier's disease.
3. The association between these diseases could also be by chance, with no true biological association, caused only because depression is a very prevalent disorder in the general population, reaching up to 13% of people during their life times. But in a study with a family with cosegregation between affective disorders and Darier's disease, Craddock et al.^2^ demonstrated that the probability of this association occurring by chance was very low^.^
4. The gene responsible for Darier's disease may be located very close to the susceptibility gene for depression, in the same chromosome region. Therefore, in a rare situation in which both genes are mutated (i.e. causing the diseases), they will always be transmitted together to the next generation (linkage phenomenon). Investigating this last hypothesis, Dawson, et al.^3^ studied patients with both Darier's disease and bipolar affective disorder, and found an allelic association with the pancreatic phospholipase A2 gene (PLA2A). However, Jacobsen, et al.^4^ showed that alterations on the gene that codes the sarcoplasmic/endoplasmic reticulum calcium-pumping ATPase (SERCA2) are implicated in the appearance of Darier's disease and neuropsychiatric manifestations. Thus, they concluded that this gene should have a pleiotropic action, being responsible for both clinical manifestations.

In summary, the apparent cosegregation in this family between Darier's disease and depression, if true, could either be the result of a gene contributing to mood disorder, in the neighborhood of the Darier's disease locus on chromosome 12, or alternatively a mutation in the Darier's disease gene resulting in a direct effect on the brain. Since depression is a common phenotype, it is most probably etiologically heterogeneous, like other common disorders such as hypertension or diabetes. Therefore, the depression described above could belong to a rare subtype of depressive disorders characterized by strong genetic susceptibility and presenting a Mendelian pattern of inheritance.
